# Differential immunogenicity between HAdV-5 and chimpanzee adenovirus vector ChAdOx1 is independent of fiber and penton RGD loop sequences in mice

**DOI:** 10.1038/srep16756

**Published:** 2015-11-18

**Authors:** Matthew D. J. Dicks, Alexandra J. Spencer, Lynda Coughlan, Karolis Bauza, Sarah C. Gilbert, Adrian V. S. Hill, Matthew G. Cottingham

**Affiliations:** 1 Jenner Institute, Old Road Campus Research Building, Roosevelt Drive, Oxford, OX3 7DQ, UK

## Abstract

Replication defective adenoviruses are promising vectors for the delivery of vaccine antigens. However, the potential of a vector to elicit transgene-specific adaptive immune responses is largely dependent on the viral serotype used. HAdV-5 (*Human adenovirus C*) vectors are more immunogenic than chimpanzee adenovirus vectors from species *Human adenovirus E* (ChAdOx1 and AdC68) in mice, though the mechanisms responsible for these differences in immunogenicity remain poorly understood. In this study, superior immunogenicity was associated with markedly higher levels of transgene expression *in vivo*, particularly within draining lymph nodes. To investigate the viral factors contributing to these phenotypes, we generated recombinant ChAdOx1 vectors by exchanging components of the viral capsid reported to be principally involved in cell entry with the corresponding sequences from HAdV-5. Remarkably, pseudotyping with the HAdV-5 fiber and/or penton RGD loop had little to no effect on *in vivo* transgene expression or transgene-specific adaptive immune responses despite considerable species-specific sequence heterogeneity in these components. Our results suggest that mechanisms governing vector transduction after intramuscular administration in mice may be different from those described *in vitro*.

Replication defective E1/E3 deleted recombinant adenoviruses have become important tools in the development of new vaccines. The first generation of vectors based on common human serotypes such as human adenovirus type 5 (HAdV-5) elicited potent and protective CD8^+^ T cell responses in preclinical studies. However, in subsequent human trials including a large Phase IIb HIV vaccine study, some vectors based on HAdV-5 have shown a lack of clinical efficacy^1^,^2^. Pre-existing vector-specific T cells and antibodies may have contributed to the lack of efficacy observed in these studies^2^,^3^. Vectors based on human adenovirus types with lower seroprevalence and non-human types including those isolated from chimpanzees have therefore been developed. Indeed, many of these alternative serotypes have elicited robust CD8^+^ T cell responses in human trials^4^,^5^,^6^,^7^,^8^,^9^ including a recent Ebola virus vaccine candidate^10^.

However, a consensus is emerging that vectors derived from rare human and chimpanzee adenoviruses (ChAds) often elicit lower transgene antigen-specific immune responses than HAdV-5 in mice, the most widely used pre-clinical model^11^,^12^,^13^,^14^,^15^. The mechanisms underlying these differences in immunogenicity remain poorly understood, hampering rational selection and development of new vector serotypes. HAdV-5 is a member of species *Human adenovirus C* (HAdV-C), while most other alternative human serotypes in clinical use are members of species HAdV-B (HAdV-35), or HAdV-D (HAdV-26, HAdV-28), and many ChAd vectors are derived from members of HAdV-E (ChAd63, AdC68, ChAdOx1). In the current study, we investigate the mechanisms responsible for the previously reported^14^ differences in immunogenicity in mice between HAdV-5 and ChAdOx1, a new HAdV-E ChAd vector shown to be safe and immunogenic in a recent human influenza vaccine trial^14^,^16^,^17^.

Higher transgene antigen-specific responses after HAdV-5 compared to AdC68 and ChAd63 vaccination have been associated with higher levels of transgene expression *in vivo*^15^,^18^. We hypothesized that differences in transgene expression could be due to differences in the efficiency of cell entry and/or trafficking to the nucleus between vectors *in vivo*. Priming and maturation of professional antigen presenting cells (APCs) after vaccination is required for antigen presentation and initiation of protective T cell and antibody responses. However, the precise cell-types preferentially transduced by adenovirus vectors, and the contributions of direct and cross-presentation of antigen to APCs after vaccination remain unclear. Although receptor usage has been extensively studied for HAdV-C adenoviruses^19^,^20^, mechanisms of HAdV-E entry remain poorly understood, though both HAdV-5 and AdC68 have been shown to utilize the coxsackie and adenovirus receptor (CAR) to enter cells *in vitro*^21^,^22^. The classical pathway of adenovirus entry involves an initial high affinity interaction between the adenovirus fiber protein and a cell surface receptor such as CAR^23^,^24^ or CD46^25^, followed by a subsequent interaction between a tri-peptide Arg-Gly-Asp (RGD) motif on the viral penton base and cellular α_v_ integrins to facilitate clathrin-dependent endocytosis^26^,^27^,^28^. Both the adenovirus fiber and the flexible penton RGD loop exhibit considerable sequence heterogeneity between viral species. In the current study, we assess the contribution of these key viral sequences to the differences in immunogenicity and transgene expression between HAdV-5 and ChAdOx1 vector vaccines, to facilitate rational design of improved vector platforms.

## Results

### HAdV-5 vaccine vectors elicit higher antigen specific immune responses and higher levels of transgene expression *in vivo* compared to ChAdOx1 vectors

**Figure 1:**
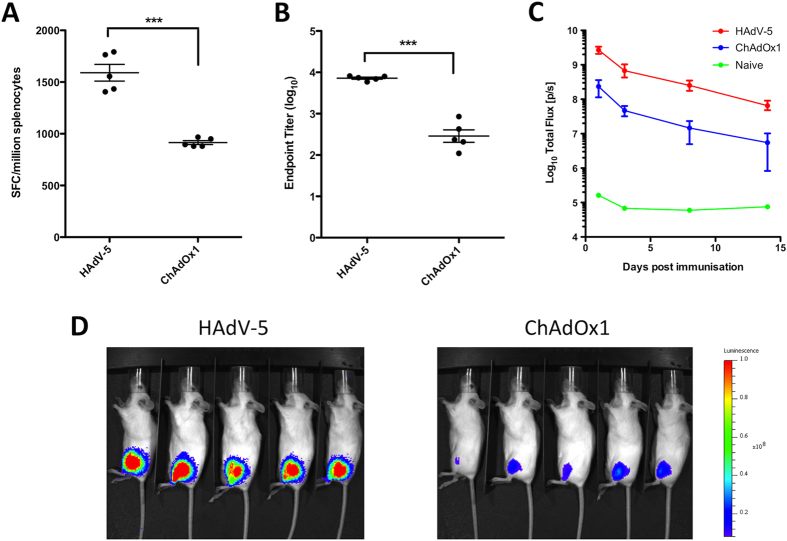
HAdV-5 vaccine vectors elicit higher CD8^+^ T cell and antibody responses, and deliver superior transgene expression *in vivo* compared to ChAdOx1 vectors. BALB/c mice (5/group) were vaccinated intramuscularly with 10^8^ infectious units (ifu) HAdV-5 or ChAdOx1 expressing *Photinus* luciferase. Two weeks post vaccination, (**A**) splenic CD8^+^ T cell responses against dominant Luc_160-168_ epitope GFQSMYTFV were measured by IFN-γ ELISpot and (**B**) anti-luciferase IgG titers were assessed in serum by endpoint ELISA. Statistical analyses performed by student T-test. (**C**) Prior to termination of the experiment, luciferase expression *in vivo* was measured in the same animals by bioluminescence imaging on day, 1, 3, 7 and 14, post vaccination. Graph shows mean total flux units per second (p/s) ± range. (**D**) Overlay image indicating the location of the bioluminescence signal 24 h post vaccination in both HAdV-5 and ChAdOx1 vaccinated groups. Intensity of signal indicated, where red is most intense (10^8^ p/s) and violet is least intense (10^7^ p/s).

The ability of HAdV-5 and ChAdOx1 vectors to induce transgene-antigen specific immune responses after intramuscular administration was comparatively assessed in mice. BALB/c mice were vaccinated with HAdV-5 or ChAdOx1 vectors expressing *Photinus pyralis* luciferase at a dose of 10^8^ infectious units (ifu). Two weeks post vaccination, splenic CD8^+^ T cell responses against dominant epitope Luc_160–168_, as assessed by IFN-γ ELISpot, were statistically significantly higher in magnitude after HAdV-5 vaccination than ChAdOx1 vaccination (Fig. 1A). HAdV-5 vaccination also elicited anti-luciferase IgG ELISA titers over 50-fold higher than after ChAdOx1 administration (Fig. 1B). Prior to sacrificing the animals, bioluminescence imaging was performed at several time points from 24 h to 14 days post vaccination after systemic administration of D-luciferin to assess luciferase transgene expression *in vivo*. Mean bioluminescent flux remained approximately 10-fold higher after HAdV-5 vaccination compared to ChAdOx1 for at least two-weeks post vaccination (Fig. 1C), and bioluminescence signal was detected almost exclusively within the injected leg 24 h post vaccination (Fig. 1D) and throughout the duration of study (data not shown). Total bioluminescent flux declined during the period of study at a similar rate after vaccination with both vectors, exhibiting an approximate 50-fold reduction between 24 h and 14 days post vaccination with both HAdV-5 and ChAdOx1 (Fig. 1C).

### HAdV-5 vectors transduce APCs more efficiently than ChAdOx1 *in vivo* after intramuscular administration

**Figure 2:**
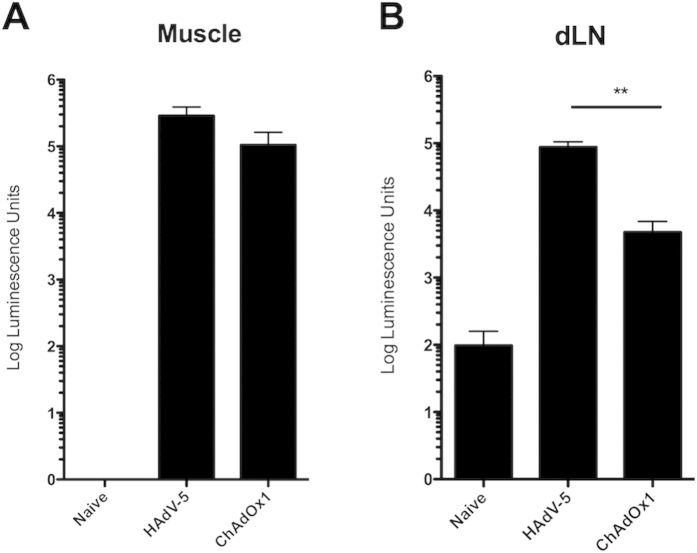
Transgene expression is higher within draining popliteal lymph nodes after HAdV-5 vaccination than ChAdOx1 vaccination. BALB/c mice, (3/group) were vaccinated intramuscularly with 10^8^ ifu HAdV-5 or ChAdOx1 vectors expressing *Photinus* luciferase. 24 h later, mice were sacrificed, and popliteal lymph nodes and muscle tissue removed for extraction of luciferase protein. Luciferase activity was assayed *in vitro* from (**A**) muscle and (**B**) lymph node extracts. Bars show mean and SEM. Statistical analysis by two-tail student t-test between HAdV-5 and ChAdOx1 vaccinated groups. Results are representative of three independent experiments.

**Figure 3:**
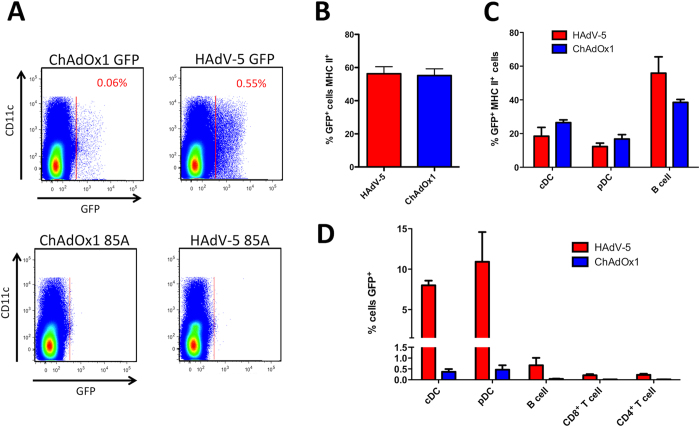
HAdV-5 vectors transduce lymph node resident antigen presenting cells more efficiently *in vivo* than ChAdOx1. BALB/c mice (3/group) were vaccinated intramuscularly with 10^9^ ifu of HAdV-5 eGFP or ChAdOx1 eGFP. A further two groups vaccinated with HAdV-5 and ChAdOx1 vectors expressing non-fluorescent Ag85A were included as a negative control. At 24 h post vaccination, proximal popliteal draining lymph nodes (dLN) were harvested and expression of GFP was assessed within live cells by flow cytometry. (**A**) Percentage of all live cells within the dLN expressing eGFP. Co-expression of surface dendritic cell (DC) marker CD11c is also plotted on the y-axis. Data shown from one representative individual in each group, and result is representative of three independent experiments. (**B**) Percentage of GFP^+^ cells (from **A**) expressing MHC Class II. (**C**) Percentage of GFP^+^ MHC II^+^ cells (from **B**) with the phenotype CD11c^+^B220^−^ (cDCs), CD11c^+^B220^+^ (pDCs) and CD11c^−^B220^+^ (**B** cells). (**D**) Total live cells from the same experiment were phenotyped and %GFP expression assessed within each subset. Graphs in **B–D** show mean and SEM of data from each individual per group.

Both direct priming of professional APCs within dLNs, and cross-priming by antigen expressed from non-hematopoietic tissues has been reported following adenovirus vector administration^29^,^30^,^31^. To further investigate the difference in luciferase expression between vectors, BALB/c mice were vaccinated with HAdV-5 or ChAdOx1 vectors expressing luciferase and muscle tissue samples from the site of injection and draining popliteal lymph nodes were harvested 24 h post vaccination. Luciferase expression was detected *ex vivo* in both muscle and lymph node tissue of vaccinated animals (Fig. 2A,B). Since the extraction procedure differed between muscle and lymph node samples, absolute luminescence units cannot be compared between tissues. However, in lymph node samples, mean luminescence activity after HAdV-5 vaccination was approximately 20-fold higher than after ChAdOx1 vaccination (Fig. 2A). By contrast, mean luminescence activity within muscle samples was less than 3-fold greater after HAdV-5 vaccination (Fig. 2B). These data suggest that the greatest differences in transduction efficiency between HAdV-5 and ChAdOx1 *in vivo* occur within the draining lymph nodes after intramuscular vaccination. To investigate which cell types are transduced within dLNs, mice were vaccinated with vectors expressing either enhanced GFP (eGFP) or a non-fluorescent control antigen (Ag85A from *Mycobacterium tuberculosis*). Lymph nodes were harvested 24 h post vaccination and cells stained with fluorophore-conjugated antibodies against surface markers. Figure 3A indicates that although GFP positive cells were detected in both HAdV-5 and ChAdOx1 groups vaccinated with GFP expressing vectors, a 10-fold higher percentage of cells were GFP positive following HAdV-5 vaccination. The intensity of the GFP fluorescent signal within infected cells was comparable. In both groups, almost 60% of these GFP positive cells were professional APCs, expressing MHC class II (Fig. 3B). Of these GFP^+^ MHC class II^+^ cells, approximately 20% were CD11c^+^B220^−^ (defined here as conventional DCs), 10% were CD11c^+^B220^+^ (defined here as plasmacytoid DCs) and between 40–50% were CD11c^−^B220^+^ (defined here as B cells) (Fig. 3C). The relative proportions of GFP^+^ cells displaying each phenotype were comparable between HAdV-5 and ChAdOx1 vaccinated groups. However, this analysis does not take into account the difference in population sizes between cells of different phenotype. There were, for instance, many more B cells within draining lymph node than CD11c^+^ DCs, with cDCs and pDCs comprising just 5% and 2% of the total MHC class II positive population respectively (data not shown). The data were re-analysed, with total cell populations of a particular phenotype identified and gated prior to analysis of GFP expression. Figure 3D indicates that a 20-fold higher percentage of CD11c^+^ cells (cDCs and pDCs) expressed GFP after HAdV-5 vaccination (~10%) compared to B cells (~0.5%). A 10-fold higher percentage of cDCs and pDCs expressed GFP after HAdV-5 vaccination than ChAdOx1 vaccination, in agreement with the relationship observed across the entire lymph node in Fig. 3A.

### Replacement of fiber and penton RGD sequences from ChAdOx1 with corresponding sequences from HAdV-5 modifies transduction efficiency *in vitro*

Our data suggest that there may be an association between the efficiency of transduction *in vivo* and immunogenicity of vaccine vectors from different adenovirus species. To investigate the vector components responsible for the observed differences in transduction and immunogenicity between HAdV-5 and ChAdOx1, fiber (stalk and knob domains) and penton RGD loop sequences were deleted from the ChAdOx1 genome and replaced with the corresponding sequences from HAdV-5 through bacterial artificial chromosome (BAC) *GalK* recombineering^32^.

The stalk and knob domains constitute the surface exposed region of the trimeric fiber protein, with the knob domain required for engagement of cell surface receptors^33^. The conserved fiber tail domain^34^, embedded in the viral capsid, was retained from ChAdOx1 in the chimeric constructs to enhance stability of the capsid, similar to an approach described previously^35^. The fiber protein has been shown to exhibit species-specific variation in sequence identity^36^, length^35^ and flexibility^37^ all of which have been shown to promote differences in tropism between members of different viral species. The amino acid sequence identity between HAdV-5 and ChAdOx1 is a modest 52% and the HAdV-5 fiber is also much longer (581 amino acids compared to 443 amino acids for ChAdOx1). Indeed, fiber proteins from species HAdV-C tend to have fiber shaft domains over twice the length of members from HAdV-E^19^,^38^. The penton base protein is highly conserved between adenoviral species with the exception of two surface-exposed loop domains, the RGD loop and hyper-variable region 1 (HVR-1)^39^,^40^. The RGD loop is a hyper-variable flexible loop containing the tri-peptide RGD motif that facilitates virus attachment to cellular integrins^27^,^28^,^34^. While members of all human adenovirus species except HAdV-F^40^ retain the key RGD motif, the length of the loop varies considerably and is almost double the size in HAdV-5 (82 amino acids) compared to ChAdOx1 (43 amino acids). At present the biological significance of sequence variability within the RGD loop is not fully understood.

**Figure 4:**
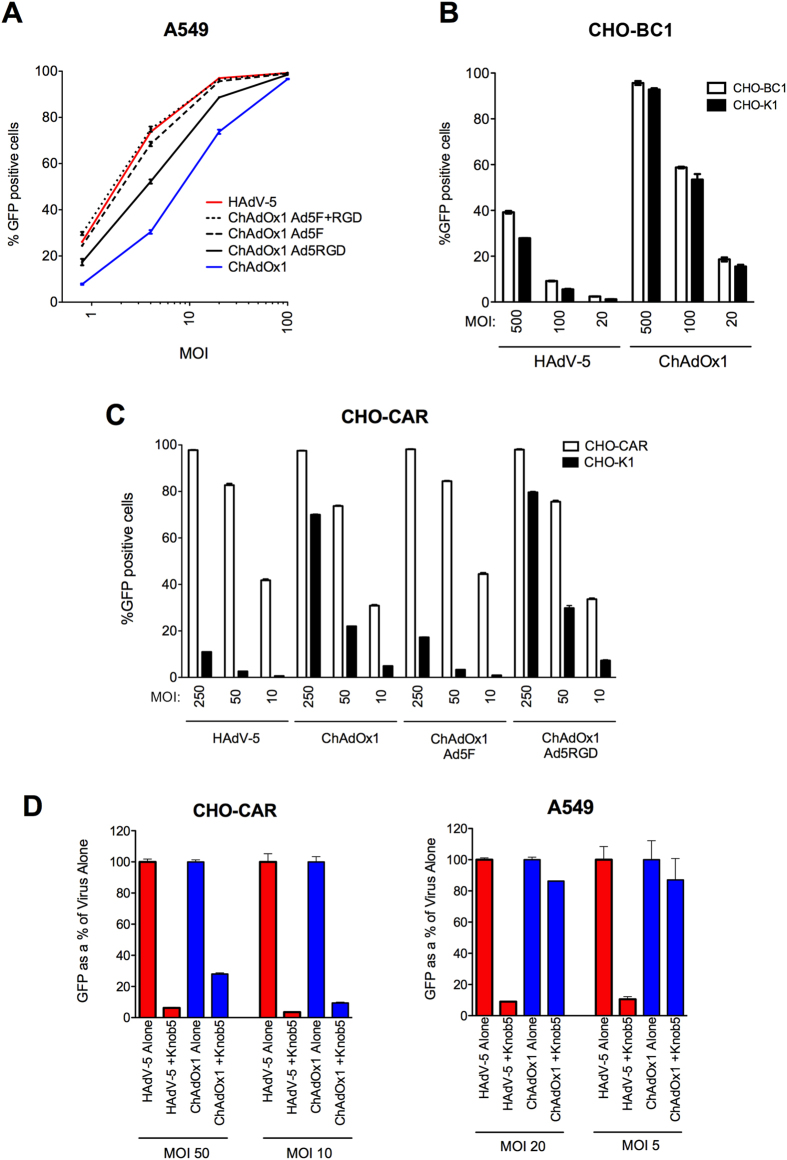
Replacement of fiber and penton RGD sequences from ChAdOx1 with corresponding sequences from HAdV-5 modifies transduction efficiency *in vitro*. (**A**) A549 cells were incubated with HAdV-5, ChAdOx1 or ChAdOx1 chimeric vectors with the HAdV-5 fiber (Ad5F) or HAdV-5 penton RGD sequences (Ad5RGD), or both HAdV-5 fiber and penton RGD sequences (Ad5F+RGD) all expressing TIPeGFP. Vectors were added at several different multiplicities of infection (MOI) as shown. 24 h post infection, the percentage of cells expressing GFP was measured by flow cytometry. (**B**) To investigate the efficiency of virus entry via surface receptor CD46, chinese hamster ovary (CHO) cells stably expressing human CD46, (CHO-BC1) and CHO cells lacking CD46 expression (CHO-K1) were incubated with HAdV-5 or ChAdOx1 vectors expressing TIPeGFP for 1 h at 4 °C. Virus was then removed and cells washed before incubation for a further 24 h and assessment of GFP expression by flow cytometry. (**C**) To investigate the efficiency of virus entry via the coxsackie and adenovirus receptor (CAR), CHO cells stably expressing human CAR (CHO-CAR) and CHO-K1 cells were incubated with HAdV-5, ChAdOx1 and ChAdOx1 chimeric vectors expressing TIPeGFP. Virus was incubated with cells at 4 °C, removed, and GFP expression measured exactly as described in (**B**). (**D**) To further investigate the contribution of CAR mediated entry during transduction of CHO-CAR and A549 cells, HAdV-5 or ChAdOx1 vectors expressing GFP were incubated with cells in the presence or absence of recombinant HAdV-5 fiber knob protein (Knob5) previously shown to inhibit CAR mediated viral entry^41^,^42^ Virus was removed, cells washed, and transduction measured as described in (**B**,**C**). All graphs show mean and SEM of triplicate wells.

Chimeric ChAdOx1 vectors with the fiber (ChAdOx1-Ad5F), penton RGD loop (ChAdOx1-Ad5RGD) and both fiber and penton RGD loop (ChAdOx1-Ad5F+RGD) sequences from HAdV-5 were constructed. The resulting vectors were sequenced across the modified region(s), and the modifications introduced no additional nucleotides other than HAdV-5 sequence. Chimeric vectors expressing GFP were successfully rescued in HEK293 cells, and transduction *in vitro* tested on human lung carcinoma A549 cells. HAdV-5 vectors transduced A549 cells with a 10-fold higher efficiency than unmodified ChAdOx1; a 10-fold lower multiplicity of infection (MOI) of HAdV-5 was required to obtain equivalent percentage transduction compared to ChAdOx1 (Fig. 4A). However, replacement of the ChAdOx1 fiber with the HAdV-5 fiber increased transduction of ChAdOx1, both in the presence and absence of additional penton modification, to the same efficiency as HAdV-5. This suggests that the difference in transduction of A549 cells between HAdV-5 and ChAdOx1 is fiber dependent. A modest improvement in transduction efficiency was also observed upon replacement of the penton RGD loop alone (ChAdOx1-Ad5RGD). Importantly, the improvement in transduction observed with all three chimeric vectors shows that the HAdV-5 fiber and RGD loop regions are functional in the context of the ChAdOx1 capsid, and that the chimeric capsids are structurally stable.

Differences in receptor usage between HAdV-5 and ChAdOx1 were investigated further. Figure 4B shows that stable expression of the CD46 surface receptor, required for cellular attachment of HAdV-B adenoviruses, has no effect on the ability of either HAdV-5 or ChAdOx1 to bind to Chinese hamster ovary (CHO) cells. In both CHO-BC1 (expressing CD46) and CHO-K1 (control) cells, efficiency of transduction by ChAdOx1 was over 5-fold higher than HAdV-5. In contrast, stable expression of the coxsackie and adenovirus receptor (CAR) increased efficiency of transduction of both HAdV-5 and ChAdOx1 vectors (Fig. 4C). In CHO-CAR cells, a modest increase in efficiency of transduction of HAdV-5 and ChAdOx1-Ad5F compared to ChAdOx1 vectors lacking the HAdV-5 fiber was observed. Interestingly, in CHO-K1 cells lacking the CAR receptor, ChAdOx1-Ad5F exhibited comparable transduction efficiency to that of HAdV-5. These data indicate that ChAdOx1 has a fiber dependent, but CAR- and CD46-independent mechanism of cell attachment to CHO-K1 cells that is not exploited by HAdV-5. To investigate the contribution of CAR-mediated entry during transduction of CHO-CAR and A549 cells, cell monolayers were pre-incubated with recombinant HAdV-5 fiber knob (Knob5) protein (shown previously to competitively inhibit CAR-dependent entry of adenovirus vectors^41^,^42^) prior to transduction (Fig. 4D). Transduction of CHO-CAR cells by both HAdV-5 and ChAdOx1 was primarily CAR-dependent, with Knob5 pre-incubation markedly reducing transduction efficiency of both vectors. While transduction of A549 cells by HAdV-5 was also CAR-dependent, transduction by ChAdOx1 was largely CAR-independent implying that an alternative route of entry for ChAdOx1 also exists in this cell line.

### Replacement of fiber and penton RGD sequences from ChAdOx1 with corresponding sequences from HAdV-5 does not increase CD8^+^ T cell or antibody responses *in vivo*

**Figure 5:**
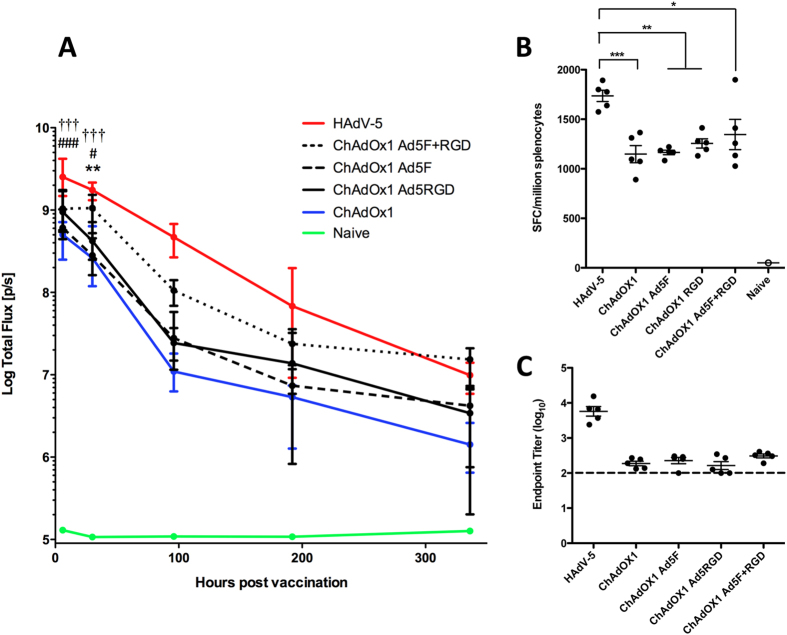
Replacement of fiber and penton RGD sequences from ChAdOx1 with corresponding sequences from HAdV-5 fails to improve magnitudes of T cell and antibody responses. BALB/c mice (5/group) were vaccinated intramuscularly with 10^8^ infectious units (ifu) HAdV-5, ChAdOx1, or ChAdOx1 chimeric vectors expressing *Photinus* luciferase. (**A**) Luciferase expression *in vivo* was measured by bioluminescence imaging 6 h, 24 h, 4 days, 8 days, and 14 days post vaccination. Graph shows mean total flux units per second (p/s) ± range. Statistical analysis performed by repeating measures two-way ANOVA with Bonferroni post test. Dagger (†) indicates statistical significance between HAdV-5 and ChAdOx1, ChAdOx1 Ad5F and ChAdOx1 Ad5RGD; hash (#) indicates significance between HAdV-5 and ChAdOx1 Ad5F+RGD; and asterisk (*) indicates significance between ChAdOx1 and ChAdOx1 Ad5F+RGD. (**B**,**C**) After 14 days post vaccination, (**B**) splenic CD8^+^ T cell responses against dominant Luc_160–168_ epitope GFQSMYTFV were measured by IFN-γ ELISpot and (**C**) anti-luciferase IgG titers were assessed in serum by endpoint ELISA. Statistical analyses in (**B**,**C**) performed by one-way ANOVA.

The ability of chimeric ChAdOx1-Ad5F, ChAdOx1-Ad5RGD and ChAdOx1-Ad5F+RGD to deliver transgene expression *in vivo* compared to parent ChAdOx1 and HAdV-5 was assessed. BALB/c mice were vaccinated intramuscularly with recombinant vectors expressing *Photinus pyralis* luciferase at a dose of 10^8^ ifu, and luciferase expression measured *in vivo* through bioluminescence imaging. In agreement with our previous data (Fig. 1), Fig. 5A indicates that bioluminescence signal remained at least 10-fold higher after HAdV-5 vaccination than ChAdOx1 for two weeks post administration. Indeed, bioluminescence signal in the HAdV-5 vaccinated group, was statistically significantly higher compared to all other groups at both 6 h and 24 h post administration. ChAdOx1-Ad5F and ChAdOx1-RGD vectors offered no statistically significant improvement in transgene expression over ChAdOx1. However, ChAdOx1-Ad5F+RGD did elicit a statistically significant improvement in transgene expression after 24 h (over 5-fold) compared to ChAdOx1. This trend continued for two weeks post vaccination, with mean bioluminescence flux remaining between 5-fold and 10-fold higher after ChAdOx1-Ad5F+RGD vaccination than ChAdOx1. Bioluminescence signal decreased with time at a similar rate in all vaccinated groups. Interestingly, in all groups, the magnitude of bioluminescence signal was at least as high 6 h post vaccination as after 24 h, indicating that peak transgene expression levels were reached very rapidly post vaccination.

After two weeks, the same mice were sacrificed and luciferase specific CD8^+^ T cell and antibody responses were assessed. HAdV-5 vaccination elicited statistically significantly higher splenic CD8^+^ T cell responses and serum anti-luciferase IgG titers than in any of the ChAdOx1 or chimeric ChAdOx1 vaccinated groups (Fig. 5B,C). None of the chimeric vectors offered a statistically significant improvement in immunogenicity compared to ChAdOx1, though there was a trend towards higher CD8^+^ T cell responses in the ChAdOx1-Ad5F+RGD vaccinated group.

**Figure 6:**
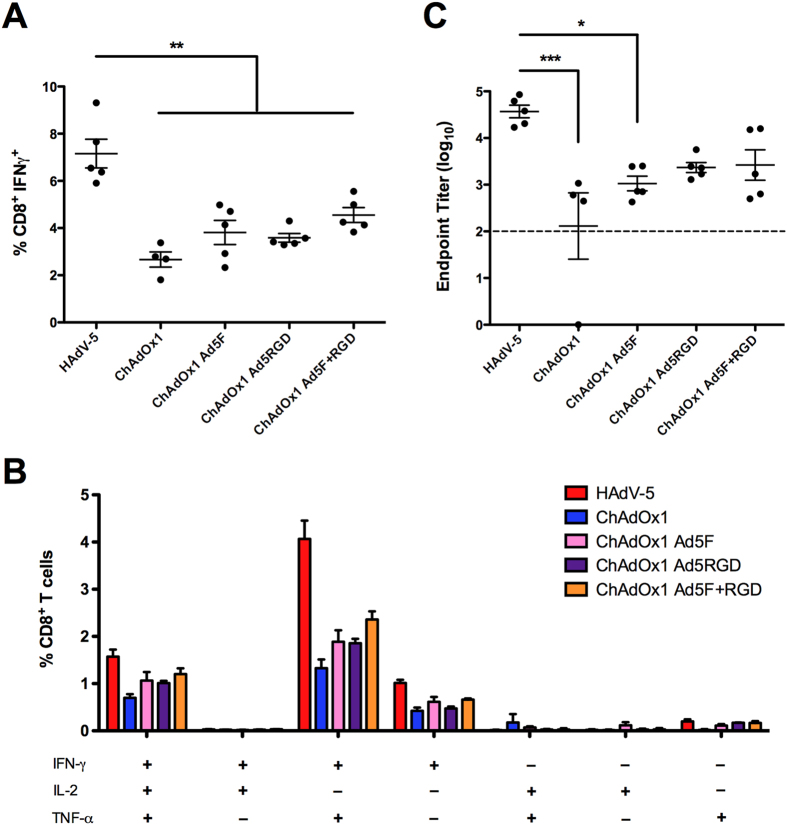
Transgene-specific adaptive immune responses are comparable between ChAdOx1 and fiber and/or penton RGD chimeric vectors expressing the TIPeGFP antigen construct. BALB/c mice were vaccinated intramuscularly with 10^8^ ifu HAdV-5, ChAdOx1 or ChAdOx1 chimeric vectors expressing TIPeGFP. After 14 days post vaccination, CD8^+^ T cell responses against immunodominant epitope *Pb*9 (in the TIP epitope string) were measured by ICS. (**A**) *Pb*9 specific IFN-γ^+^CD8^+^ T cells as a percentage of total CD8^+^ T cells. (**B**) Percentages of *Pb*9 specific CD8^+^ T cells secreting combinations of the three cytokines IFN-γ, IL-2 and TNF-α. (**C**) Anti-GFP IgG titers in serum measured by endpoint ELISA. Dashed lines in (**C**) indicate limit of detection of the assay. Statistical analyses in (**A**,**C**) performed by one-way ANOVA.

In a separate experiment, BALB/c mice were vaccinated with the same series of chimeric vectors expressing a different recombinant antigen construct, TIPeGFP (Fig. 6). Despite a trend towards a modest improvement, no statistically significant increases in splenic IFNγ^+^CD8^+^ T cell frequencies or serum anti-GFP IgG titers were observed with the chimeric vectors compared to ChAdOx1 (Fig. 6A,C). Cytokine production profiles for IFN-γ, IL-2 and TNF-α by transgene product-specific CD8^+^ T cells were comparable between ChAdOx1 and the chimeric vectors (Fig. 6B). The data also show that most IFN-γ^+^CD8^+^ T cells elicited by HAdV-5 vaccination also produce TNF-α.

### Distribution of transgene expression within lymph node and muscle tissue differs after intramuscular vaccination with HAdV-5, ChAdOx1, and ChAdOx1-Ad5F+RGD

**Figure 7:**
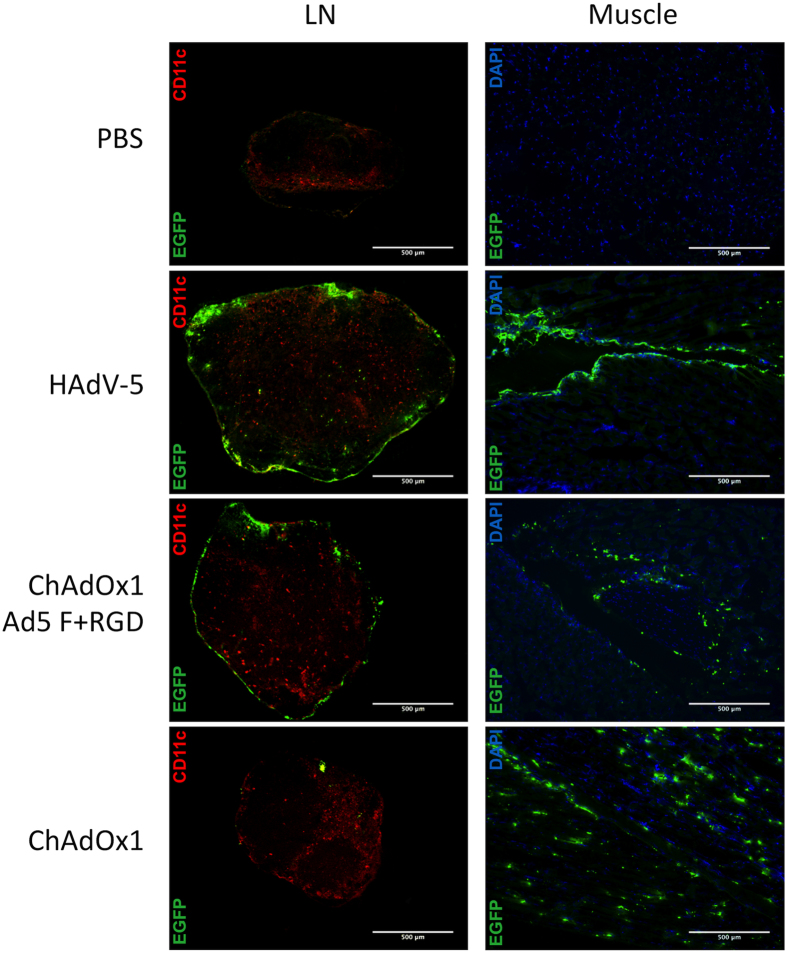
Distribution of transgene expression within lymph node and muscle tissue differs after vaccination with HAdV-5 and ChAdOx1. BALB/c mice were vaccinated intramuscularly with 10^9^ ifu HAdV-5 TIPeGFP, ChAdOx1 TIPeGFP, ChAdOx1 Ad5F+RGD TIPeGFP, or PBS alone. After 24 h, popliteal dLNs and samples of muscle tissue from the site of injection were collected and mounted in OCT compound for cryo-sectioning and immunohistochemistry. LN tissue was stained for localization of GFP (green) and CD11c (red) expression. Co-localisation of GFP and CD11c is shown in yellow. Muscle tissue was stained with DAPI (blue) and for localization of GFP (green) expression. Images (all at magnification 5x) are from one representative individual from each group (n = 3).

To gain a more detailed insight into the localization of transgene expression, draining popliteal LNs and muscle tissue from the site of injection were collected 24 h post vaccination for cryo-sectioning. LN tissue was stained for expression of DC surface marker CD11c, and muscle tissue was stained with nuclear stain DAPI. Both tissues were stained for GFP expression. In LNs, distribution of GFP expression was far more extensive after HAdV-5 vaccination than ChAdOx1 vaccination (Fig. 7), in agreement with our previous observations (Figs 2 and 3). After vaccination with ChAdOx1-Ad5F+RGD, distribution of GFP expression within dLNs was comparable to HAdV-5. After vaccination with HAdV-5 or ChAdOx1 Ad5F+RGD, GFP signal was localized to tissue at the anatomical edge of the lymph node, potentially the sub-capsular sinus. There was some degree of co-localisation of GFP signal with CD11c after HAdV-5 vaccination, but for the most part GFP expression did not strongly co-localise with CD11c expression. In muscle, the difference in abundance of GFP expression between vectors was less clear, but there was a marked difference in the localization of signal (Fig. 7). After HAdV-5 vaccination, GFP expression was largely confined to the outer surface of muscle fibers, while after ChAdOx1 and ChAdOx1-Ad5F+RGD vaccination GFP expression was dispersed throughout the muscle tissue. No GFP signal was observed in the LNs and muscle of PBS injected control animals.

## Discussion

In mice, rare serotype adenovirus vectors from HAdV-B, HAdV-D and HAdV-E tend to elicit lower transgene antigen specific cellular and humoral immune responses than common human serotype HAdV-5 (HAdV-C). Data from this study (Fig. 1) and others^15^,^18^, have associated differences in immunogenicity between HAdV-5 and HAdV-E types with marked differences in the efficiency of transgene delivery *in vivo* post administration.

We sought to determine the nature of cell and tissue types transduced by HAdV-5 and ChAdOx1 after intramuscular delivery. While professional APCs in the draining lymph node (dLN) are required for initial priming, studies from the Bramson laboratory have shown that persistent antigen presentation by non-hematopoetic APCs outside the dLN, potentially within muscle or endothelial cells at the site of injection, is required to sustain CD8^+^ T cell memory^43^,^44^. In this study, there was no difference in transgene expression within muscle tissue at the injection site between mice vaccinated with HAdV-5 and ChAdOx1. There was however a marked difference in transgene expression within cells in the dLNs proximal to the site of injection. This was observed through *ex vivo* analysis of luciferase expression in lymph nodes and muscle (Fig. 2), and immunohistochemistry of muscle and lymph node sections from vaccinated animals (Fig. 7).

Flow cytometric analysis of cells expressing transgene from dLNs post-vaccination, also supported the observation that transgene expression within dLNs is approximately 10-fold higher after HAdV-5 administration, though there was little difference in the phenotypes of transgene-positive cells between groups (Fig. 3). Stable fluorescent signal within these cells is assumed (but not directly shown) to have derived from *de novo* GFP expression following direct vector transduction^29^ rather than through phagocytosis of extracellular GFP, implying that direct antigen presentation is more efficient after HAdV-5 vaccination. The increased antigen expression within multiple DC subsets *in vivo* after HAdV-5 vector vaccination observed here is in agreement with a recent study^15^ and demonstrates that HAdV-5 viruses have an increased propensity to enter APCs, or to express transgene within infected APCs that is not specific to a particular cell subset. A significant percentage of transgene-positive cells in both HAdV-5 and ChAdOx1 vaccinated groups were B cells, although GFP^+^ cells represented less than 1% of the total B cell population. The importance of B cell transduction in the context of antigen presentation may be limited, since B cells are not efficient antigen presenters and CD8^+^ T cell priming has been shown to require CD4^+^ T cell help^45^. In contrast, roughly 10% of cDCs and pDCs were GFP^+^ after HAdV-5 vaccination, while the ChAdOx1 vector transduced less than 1% of either cell type (Fig. 3). Immunohistochemistry on lymph node tissue (Fig. 7) shows that GFP expression was primarily localized to the periphery of the node after HAdV-5 vaccination, potentially the subcapsular sinus (SCS), and did not strongly co-localise with CD11c. It may be that other cell types such as SCS macrophages, or endothelial cells are important repositories for antigen expression after HAdV-5 vaccination^46^,^47^,^48^.

Through reverse genetics, we investigated whether differences in sequence identity between vectors in the fiber and penton RGD loop regions could be responsible for the observed differences in immunogenicity and transgene expression, since these capsid surface structures are essential for mediating viral entry into cells *in vitro*. Previous studies in mice have shown that exchanging fiber proteins between HAdV-5 and HAdV-35 serotypes can alter vaccine immunogenicity^49^,^50^. In addition to the nature of the receptor-fiber knob interaction, there is evidence though studies with HAdV-C, D and B chimeras, that the length^35^ and flexibility^37^ of the fiber shaft can also influence binding and infectivity. Furthermore, fiber pseudo-typing has been shown to alter transduction and inflammatory profiles of vectors *in vivo*^51^,^52^. The penton RGD loop, in addition to its role in cellular entry, has also been shown to be important for the induction of proinflamatory cytokines such as IL-1α upon viral infection^53^. Most studies involving the adenovirus penton have involved direct mutation of the RGD motif rather than its context within the hyper-variable loop, though a recent publication has shown that pseudo-typing of the HAdV-5 capsid with both fiber and penton from HAdV-35 (but not fiber alone) can improve transduction of human smooth muscle tissue^54^.

All three chimeric ChAdOx1 vectors, ChAdOx1-Ad5F, ChAdOx1-Ad5RGD, and ChAdOx1-Ad5F+RGD transduced A549 cells with greater efficiency than the parent ChAdOx1 vector (Fig. 4). Replacement of the fiber alone was sufficient to confer equivalent transduction between HAdV-5 and ChAdOx1 vectors in this cell line. In agreement with previous observations^21^, stable expression of the CAR receptor increased transduction of CHO cells for both HAdV-5 and ChAdOx1. However, the relevance of CAR mediated entry after intramuscular administration *in vivo* has been questioned, since expression is absent in muscle tissue^23^, on dendritic cells^55^, and is restricted to tight junctions between epithelial cells^56^. Cell attachment was CD46-independent for both HAdV-5 and ChAdOx1 in common with most (but not all^57^,^58^) non-HAdV-B viruses. However, binding studies with CHO cells expressing neither CAR nor CD46 suggest that ChAdOx1 has an additional fiber-dependent mechanism of entry that is not shared by HAdV-5. Furthermore, CAR receptor blocking studies suggest that transduction of A549 cells by ChAdOx1 is also primarily CAR-independent. Additional entry pathways may involve an interaction with an alternative receptor such as sialic acid, as has been demonstrated for some HAdV-D viruses^59^, or heparan sulphate proteoglycans^60^.

Despite marked differences in transduction being observed *in vitro*, replacement of fiber and penton RGD sequences failed to improve immunogenicity or transduction efficiency of ChAdOx1 *in vivo* (Figs 5 and 6). Though chimeric ChAdOx1-Ad5F+RGD exhibited improved luciferase expression compared to parent ChAdOx1 at 24 h post vaccination, expression levels were still statistically significantly lower than after HAdV-5 vaccination. Since there was also a trend towards improved CD8^+^ T cell responses after ChAdOx1-Ad5F+RGD, these data may reflect the fact that transgene expression from the chimeric constructs simply failed to reach a threshold level required to achieve a statistically significant improvement in immunogenicity.

If transgene expression does indeed correlate with efficiency of gene delivery *in vivo*, the data point to a mechanism of transduction that is predominantly independent of the nature of fiber and penton RGD sequences. The observation that an apparent 10-fold difference in transduction efficiency between HAdV-5 and ChAdOx1 exists for multiple cell types *in vivo* (Fig. 3) would also be consistent with the difference in transduction efficiency being receptor independent. Differences in the biophysical properties of the remainder of the capsid may be important^61^. It has been shown that scavenger receptors expressed on macrophages and dendritic cells have a propensity to recognize macromolecules with a negative surface charge, and targeted shielding of the HAdV-6 hexon hypervariable regions (HVR) by polyethylene glycol modification reduces uptake by Kupffer cells *in vivo*^62^. Indeed, the hexon proteins of HAdV-C viruses harbour a more negative surface charge than HAdV-E viruses^61^.

We have not excluded the possibility that soluble host factors such as coagulation factor X (FX) may also be involved in the differential transduction efficiency observed between vector serotypes. FX has been shown to mediate hepatic transduction via bridging interactions between heparan sulphate proteoglycans (HSPGs) and the viral hexon following intravenous delivery of certain adenoviral species^63^,^64^,^65^,^66^. However, the FX mediated enhancement in transduction was not observed following intramuscular delivery, suggesting that this mechanism is unlikely to be responsible for the differences in transduction observed here^63^. A recent study has suggested that the FX:hexon interaction may also play a role in protecting the HAdV-5 virion from opsonization and neutralization via host IgM and complement following intravenous delivery^67^. However, subsequent studies have demonstrated that this complement-mediated neutralization in the absence of FX is specifically directed against hypervariable regions of the HAdV-5 hexon and is not conserved amongst other adenoviral species^68^.

An alternative hypothesis might be that differences in transgene expression between vectors are independent of transduction efficiency, and are instead due to differences in the levels of transcription or translation of the encoded transgene. Indeed, type I interferon has been shown to downregulate activity of the CMV promoter^69^, and adenovirus vectors from HAdV-B, HAdV-D, and HAdV-E induce significantly more type I interferon than HAdV-5^13^,^15^,^18^. Interferon signaling could also mediate innate immune clearance of transgene expressing cells through activation of NK cells^70^. Recent studies using mice lacking the interferon-αβ receptor (*Ifnabr*^−/−^) have associated type I interferon signaling with a reduction of transgene expression after vaccination with members of these alternative viral species^13^,^15^. However, the association between interferon signaling and transgene-specific CD8^+^ T cell frequency is less clear. In a recent publication, *Ifnabr*^−/−^ mice exhibited an accelerated CD8^+^ T cell kinetic after vaccination with HAdV-E vector ChAd63^15^, but not a significantly greater peak magnitude as had previously been shown for HAdV-B and HAdV-D vectors^13^. The adenovirus derived pathogen-associated molecular patterns (PAMPs) responsible for interferon production in these studies remain to be identified, though the study by Quinn *et al*. implicated recognition of adenoviral DNA through the cytosolic sensor STING (stimulator of interferon genes), speculating that differences in the rate of viral capsid uncoating between vectors could be responsible^15^.

In summary, this study has demonstrated that considerable species-specific variation in sequence identity of the primary receptor targeting capsid components, fiber and penton RGD loop, is not responsible for differences in immunogenicity and transgene expression observed in mice between HAdV-5 and ChAdOx1. Data from this study and others imply that mechanisms involved in vector transduction *in vivo* may be different and/or considerably more complex compared to those described *in vitro*. Further investigation into the mechanisms underlying the potency of these clinically important vaccine vectors will therefore be essential for future development of this promising technology.

## Materials and Methods

### Cells and Viruses

The construction of E1-E3 deleted chimpanzee adenovirus vector ChAdOx1 has been described previously^17^. Chimeric ChAdOx1 vectors were generated through *GalK* recombineering, where *GalK* is employed in both positive and negative selection^32^. To generate ChAdOx1-Ad5F, amino acid residues 52-443 of the ChAdOx1 fiber (fiber shaft and knob domain) were deleted and replaced with the corresponding residues (52-581) from HAdV-5 fiber. Fiber tail residues (1–51) were retained from ChAdOx1 to minimize disruption of the capsid structure. To generate ChAdOx1-Ad5RGD, a 42 amino acid region corresponding to the ChAdOx1 penton RGD loop (residues 298–340 of ChAdOx1 penton) was deleted and seamlessly replaced with the 83 amino acid HAdV-5 penton RGD loop (residues 296-378 of HAdV-5 penton). Vaccine antigen constructs *Photinus* luciferase (Luc), enhanced GFP (eGFP) and TIPeGFP were cloned into the E1 locus between hCMV promoter and BGH poly A sequences. The design of vaccine antigen construct TIPeGFP (an epitope string, TIP, fused to the N-terminus of enhanced GFP) has been described previously^14^,^71^,^72^. Propagation and titration of E1-E3-deleted recombinant HAdV-5, and ChAdOx1 on HEK293A cells (Invitrogen) has been described previously^17^. The ratios of estimated viral particles to infectious particles (P:I ratios) were as follows: HAdV-5 eGFP 5, ChAdOx1 eGFP 12, HAdV-5 TIPeGFP 16, ChAdOx1 TIPeGFP 14, ChAdOx1-Ad5F TIPeGFP 22, ChAdOx1-Ad5RGD TIPeGFP 36, ChAdOx1-Ad5F+RGD TIPeGFP 26, HAdV-5 Luc 20, ChAdOx1 Luc 135, ChAdOx1-Ad5F Luc 202, ChAdOx1-Ad5RGD Luc 262, ChAdOx1 Ad5F+RGD Luc 340. P:I ratios were higher for luciferase expressing vectors since these were titered using an anti-hexon immunostaining assay rather than by direct visualization of transgene expression by fluorescence microscopy^17^. Both *in vitro* and *in vivo* experiments were performed with titers based on infectious units (IU). *In vitro* viral vector transduction assays were performed in 24-well plates as described in figure legends. To investigate receptor usage, viral vector was added to cells at 4 °C to permit binding but not internalisation of virions. After 1 h, unbound virus was removed by washing, and cells were incubated at 37 °C to enable bound virus to become internalized. For coxsackie and adenovirus receptor (CAR) blocking studies, an excess (5μg) of recombinant knob protein from HAdV-5 (Knob5) was added to 1 × 10^5^ cells for 1 h at 4 °C to inhibit CAR-mediated viral entry^41^,^42^ prior to addition of virus for a further 1 h at 4 °C. CHO-K1 cells, and CHO cells stably expressing CAR (CHO-CAR) were provided by George Santis (Kings College London). CHO cells expressing the BC1 isoform of CD46 (CHO-BC1) were obtained from Andrew Baker, University of Glasgow.

### Mice and immunization

Female BALB/c mice (Harlan, UK) above 6 weeks of age were immunized intramuscularly (i.m.). Viral vectors were formulated in phosphate buffered saline (PBS, Sigma, UK) in a total volume of 50 μl and injected into the tibialis anterior muscle of each animal. All mouse procedures were performed in accordance with the terms of the UK Animals (Scientific Procedures) Act under Home Office approved project licenses PPL 30/2414 or PPL 30/2889.

### Mouse immunology

Spleen *ex vivo* interferon-gamma (IFN-γ) ELISpot and intracellular cytokine staining (ICS) for IFN-γ, IL-2 and TNF-α was performed as described previously^14^,^17^,^73^. To measure vaccine antigen specific CD8^+^ T cell responses, cells were re-stimulated with peptides Luc_160–168_ (GFQSMYTFV)^74^ or *Pb*9 (SYIPSAEKI)^17^ at a final concentration of 1 μg/mL. IgG endpoint ELISA was performed as described previously^75^,^76^ except plates were coated with recombinant GFP protein (Millipore, UK) or recombinant *Photinus* luciferase protein (Sigma, UK) at 1 μg/mL.

### *Ex-vivo* luciferase assay

Mice were sacrificed and draining popliteal lymph nodes and tibialis anterior muscles excised. Lymph nodes were washed in PBS, crushed and cells were recovered by passage through a 70 μm strainer. Cells were lysed in NP-40 lysis buffer to release luciferase for *ex vivo* detection. Tibialis anterior muscles were macerated in NP-40 lysis buffer with glass beads using a Mini Beadbeater (Biospec Products) at maximum speed (4800 oscillations min^−1^). Homogenised material was centrifuged at 4000 rpm for 4 min, and supernatant collected. Supernatants from lymph nodes and muscle extracts were assayed for Photinus luciferase activity using the BrightGlow^TM^ Luciferase Assay System (Promega, UK) according to manufacturers instructions. Luminescence was measured using a Varioskan Flash luminometer (Thermo Scientific).

### *In vivo* bioluminescence imaging

BALB/c mice were injected with viral vectors expressing *Photinus* luciferase. Prior to imaging, animals were anaesthetised with isofluorane before subcutaneous injection of 0.75 mg D-Luciferin (Caliper Life Sciences, UK) into the scruff of the neck. Animals were kept under anesthesia for a further 8 min to allow dissemination of the luciferin substrate before image capture on an IVIS200 imaging system (Caliper Life Sciences, UK). Exposure times were adjusted to avoid oversaturation of pixels and flux measurements converted to photons per second for comparative assessment of luminescence at different time-points. Luminescence image data was analysed using Living Image 4.0 software.

### Analysis of mouse dendritic cell subsets by flow cytometry

Popliteal draining lymph nodes were processed as described previously and cells were stained with rat anti-mouse fluorochrome labelled monoclonal antibodies; CD11c-PE, CD3-PerCP-Cy5.5, B220-PECy7, MHC class II-PB, CD11b-A700, CD8-APC-A780, and CD4-efluor650 in the presence of anti-CD16/CD32 (Fc Block). All fluorochrome-conjugated antibodies were obtained from eBiosciences. Cell viability was assessed using LIVE/DEAD aqua stain (Invitrogen, UK). Expression of GFP (FITC channel) after vaccination with viral vectors encoding eGFP was assessed within live cells by flow cytometry. Data were acquired on an LSR II flow cytometer (BD) and analysed using FlowJo (Treestar).

### Cryo-sectioning and immunohistochemistry

Muscle and lymph node tissue samples were embedded in OCT compound (Sakura EU) and frozen on dry ice, prior to sectioning on a Leica CM-3050 cryostat. 6 μm sections were fixed in ice-cold acetone, rinsed in PBS, and blocked with 10% normal goat serum for 30min. Sections were incubated with primary anti-GFP rabbit polyclonal (Abcam, a290) and anti-CD11c [N418] armenian hamster monoclonal (Abcam, ab33483) for 1 h, before washing and staining with secondary Goat-anti Rabbit AlexaFluor488 and Goat anti-hamster AlexaFluor546 (Invitrogen). Samples were mounted with Prolong Gold antifade containing DAPI (Life Technologies). Images were captured on a Leica DMI3000 microscope.

## Additional Information

**How to cite this article**: Dicks, M. D. J. *et al*. Differential immunogenicity between HAdV-5 and chimpanzee adenovirus vector ChAdOx1 is independent of fiber and penton RGD loop sequences in mice. *Sci. Rep*. **5**, 16756; doi: 10.1038/srep16756 (2015).
